# Reliability of Total Serum IgE Levels to Define Type 2 High and Low Asthma Phenotypes

**DOI:** 10.3390/jcm12175447

**Published:** 2023-08-22

**Authors:** Giuseppe Guida, Francesca Bertolini, Vitina Carriero, Stefano Levra, Andrea Elio Sprio, Martina Sciolla, Giulia Orpheu, Elisa Arrigo, Stefano Pizzimenti, Giorgio Ciprandi, Fabio Luigi Massimo Ricciardolo

**Affiliations:** 1 Severe Asthma and Rare Lung Disease Unit, San Luigi Gonzaga University Hospital, Orbassano, 10043 Turin, Italy; s.pizzimenti@sanluigi.piemonte.it (S.P.); fabioluigimassimo.ricciardolo@unito.it (F.L.M.R.); 2 Department of Clinical and Biological Sciences, University of Turin, Orbassano, 10043 Turin, Italy; francesca.bertolini@unito.it (F.B.); vitina.carriero@unito.it (V.C.); stefano.levra@unito.it (S.L.); martina.sciolla@unito.it (M.S.); giulia.orpheu@unito.it (G.O.); elisa.arrigo@unito.it (E.A.); 3 Department of Research, ASOMI College of Sciences, 19112 Marsa, Malta; ae.sprio@gmail.com; 4 Allergy Clinic, Casa di Cura Villa Montallegro, 16145 Genoa, Italy; gio.cip@libero.it; 5 Institute of Translational Pharmacology, National Research Council (IFT-CNR), Section of Palermo, 90146 Palermo, Italy

**Keywords:** total serum IgE, asthma, phenotype, Type-2 inflammation, biomarkers, T2-high asthma, T2-low asthma, comorbidities, logistic analysis

## Abstract

*Background:* High total IgE levels are weak predictors of T2_High_ and have been reported in nonallergic asthma. Therefore, the role of total serum IgE (IgE) in the T2_High_ phenotype is still debated. Objective: This study investigated the reliability of stratifying asthmatics into IgE_High_ and IgE_Low_ within the T2_High_ and T2_Low_ phenotypes. *Methods:* This cross-sectional single-center study investigated the association of clinical, functional, and bio-humoral parameters in a large asthmatic population stratified by IgE ≥ 100 kU/L, allergen sensitization, B-EOS ≥ 300/µL, and F_E_NO ≥ 30 ppb. *Results:* Combining T2 biomarkers and IgE identifies (1) T2_Low_-IgE_Low_ (15.5%); (2) T2_Low_-IgE_High_ (5.1%); (3) T2_High_-IgE_Low_ (33.6%); and T2_High_-IgE_High_ (45.7%). T2_Low_-IgE_Low_ patients have more frequent cardiovascular and metabolic comorbidities, a higher prevalence of emphysema, and higher LAMA use than the two T2_High_ subgroups. Higher exacerbation rates, rhinitis, and anxiety/depression syndrome characterize the T2_Low_-IgE_High_ phenotype vs. the T2_Low_-IgE_Low_ phenotype. Within the T2_High_, low IgE was associated with female sex, obesity, and anxiety/depression. *Conclusions:* High IgE in T2_Low_ patients is associated with a peculiar clinical phenotype, similar to T2_High_ in terms of disease severity and nasal comorbidities, while retaining the T2_Low_ features. IgE may represent an additional biomarker for clustering asthma in both T2_High_ and T2_Low_ phenotypes rather than a predictor of T2_High_ asthma “*per se*”.

## 1. Introduction

Asthma is a heterogeneous disease concerning onset, natural course, and response to treatment [1]. 

Demographic, clinical, pathophysiological, and genetic characteristics of asthmatics guided the identification of recognizable clusters termed «asthma phenotypes» [2]. This approach fosters patient-tailored, efficient strategies to reduce the socio-economic asthma burden. Biomarkers measure biological or pathogenic processes (endotypes) to identify asthma phenotypes [3]. 

Asthma includes type-2 high (T2_High_) and low (T2_Low_) airway inflammation phenotypes [1,4]. Type-2 cytokine-driven eosinophilic inflammation is common (50–60%) in adults, albeit variable according to the proportion of inhaled corticosteroid (ICS)-treated patients [5]. Sputum eosinophil (S-EOS) count alone, generally >3%, is insufficient for differentiating between T2_High_ and T2_Low_ phenotypes [6]. Surrogate biomarkers, easily applicable compared with S-EOS, have been widely applied in asthma phenotyping and investigated as pathogenic-related molecules [7,8]. Peripheral eosinophils (B-EOS), total serum IgE, and fractional exhaled nitric oxide (F_E_NO) are routinely used for identifying the type-2 asthma phenotype. Generally, the assessment of FENO < 25 ppb has a higher negative predictive value for likely eosinophilic airway inflammation in asthma. However, both F_E_NO and B-EOS often lack specificity [9], as they do not completely segregate type-2 asthma phenotypes even when combined. 

Immunoglobulin E (IgE) is a protein involved in the immune response. In particular, IgE is known for its role in allergic reactions. Allergen-specific IgE binds the high-(FcεRI) and low-affinity receptors (FcεRII) for IgE located on effector cells and antigen-presenting cells. The FcεRI is constituted by α, β, and γ subunits (FcεRIα, FcεRIβ, and FcεRIγ). FcεRIα is IgE receptor-specific, whereas FcεRIβ and FcεRIγ are also part of other Fc receptors [10]. 

Allergy is per se a type-2 biomarker [11]. Allergen-specific serum IgE can generally account for up to 20–25% of total serum IgE, determining the overall value of total blood serum IgE [10]. Although total serum IgE is not a marker of asthma severity or airflow limitation, elevated levels of IgE may be associated with severe subgroups [12]. A possible role of the serum-specific IgE/total IgE ratio in predicting the clinical relevance of an allergen exposure or response to allergen immunotherapy is still under investigation.

Total serum IgE levels are usually higher in allergic versus nonallergic asthma, but they may substantially overlap. One study described three clusters of allergic asthma, with cluster 1 having a higher proportion of patients with total serum IgE levels < 100 (kU/L) (31.5%) compared with the others [13]. The Spanish MEGA cohort study did not identify total serum IgE levels as a distinctive characteristic among the four clusters described [14]. 

High IgE levels have, conversely, been extensively reported in nonallergic asthma. The bronchial mucosa of nonallergic asthma includes not only a similar expression of T2 cytokines and eosinophils as that of allergic asthma but also a similar number of FCεR1β positive cells [15]. Polymorphisms in FCεR1β have been associated with elevated total and specific IgE levels. In addition, elevated expression of local ε germline transcript (Iε) and the ε heavy chain of IgE (Cε) in the bronchial mucosa of both allergic and nonallergic asthmatics suggests local IgE synthesis [16]. How mucosal IgE production influences total serum IgE levels and if it correlates with disease activity is not known.

The usefulness of total serum IgE in differentiating allergic and nonallergic asthma is not so well defined compared with other T2 diseases, such as atopic dermatitis [17].

Therefore, it can be generally assumed that IgE is a common product of the type-2 inflammation pathway, shared by both allergic and nonallergic asthma. Unfortunately, the sensitivity and specificity of total serum IgE as a predictor of airway eosinophilia are quite weak and significantly lower than those of B-EOS and F_E_NO [18].

To date, total serum IgE is approved as the biomarker for determining eligibility in severe asthma patients for omalizumab (a humanized recombinant monoclonal antibody with binding specificity at the FcεRI binding site of IgE) treatment [19]. 

The other currently used biologics in severe asthma treatment are represented by monoclonal antibodies directed towards the main effectors of T2 inflammation: mepolizumab, an anti-IL-5 antibody; benralizumab, an anti-IL-5R antibody; and dupilumab, a fully human monoclonal antibody against the IL-4Rα subunit of the IL-4/13 receptor [20]. The modulatory effects that these biologicals can provoke on IgE concentrations are still to be clarified [21]. 

A post-hoc analysis of moderate-to-severe asthma showed a high degree of overlap among allergen-specific IgE (positive skin prick and/or serum > 0.35 kU/L), B-EOS ≥ 300 cells/μL, and F_E_NO (≥35 ppb) [22]. In this scenario, the International Severe Asthma Registry (ISAR) study reported that the likelihood of total serum IgE > 75 kU/L for the possible concomitant elevation of another biomarker was 59% for B-EOS > 300 and 65% for F_E_NO > 25 ppb [9]. Interestingly, 14% of the whole cohort reported elevated total serum IgE as a single T2_High_ marker, but only 52% and 12% of them had allergic rhinitis or atopic dermatitis, respectively. In a Danish study, the single elevation of total serum IgE of >150 IU/mL (reported in 18% of patients) was consistent with higher S-EOS in only 35%, being the majority associated with a neutrophilic or paucigranulocytic phenotype (T2_Low_) [23].

The T2_Low_ phenotype has been extensively described in asthma, often characterized as non-eosinophilic asthma, accounting for slightly more than 50% in both steroid-naïve and steroid-treated patients [24] and variably associated with poor corticosteroid response [25]. Mean total serum IgE has been reported within a range of 84 to 126 kU/L and from 78 to 107 kU/L in paucigranulocytic and neutrophilic asthma, respectively [24,26]. A more recent study analyzed the longitudinal variability of sputum granulocytes and reported a low eosinophil group with a median total serum IgE level of 114.9 kU/L [27].

These data suggest the limited value of total serum IgE as a biomarker predictive of allergic or type-2 inflammation in asthma. This study aimed to replicate, in a cross-sectional, single-center, large asthmatic cohort, the characteristics of IgE_High_ and IgE_Low_ subpopulations in terms of demographic, clinical, and functional traits and to compare them within the commonly recognized T2_High_ and T2_Low_ phenotypes. We also reasoned that high total serum IgE levels may be detected in non-allergic states, e.g., obesity, viral infections, air pollution, or smoking [28].

## 2. Materials and Methods

### 2.1. Patients and Study Design

In the current observational cross-sectional real-life study, we consecutively enrolled adults (age ≥ 18 years) as outpatients with guideline-validated asthma diagnoses [1,29], attending the “Severe Asthma Centre” of the San Luigi Gonzaga University Hospital (Orbassano, Turin, Italy) between January 2018 and December 2022. The patients signed informed consent to participate in this study. The San Luigi Gonzaga University Hospital Ethical Review Board approved the study (protocol number: 4478/2017) in accordance with the Declaration of Helsinki.

### 2.2. Collection of Clinical, Functional, and Bio-Humoral Parameters

Demographic data, clinical history, functional and humoral parameters, and medications were retrieved from chart data for each patient after at least a complete assessment, adjustment, and review management cycle, according to GINA guidelines [1]. Data were not collected in cases of current or recent asthma exacerbations (AEs), self-reported poor adherence (<50%) to treatment, or inadequate inhalation technique. A subset of severe asthmatics was divided into those who had already started a biologic (biologics ongoing) and those who were candidates to start a biologic treatment (pre-biologics). In order to avoid the influence of biologic treatment, B-EOS, total serum IgE, pulmonary function tests, and F_E_NO were collected before treatment initiation for severe asthma patients on biologics. Parasite infestation was not ruled out due to the low prevalence in Italy. Patients enrolled in the study did not have active cancer disease and had to be cancer disease-free (remission) for at least 5 years.

Pulmonary function and lung volumes were assessed by spirometry, performed before and 15 min after albuterol administration (400 µg), and plethysmography using a Vmax Encore 62 (Carefusion, Höechberg, Germany). F_E_NO was analyzed at a flow rate of 50 mL/s with FeNO+ (Medisoft, Sorinnes, Belgium). Asthma Control Test (ACT) questionnaire was used to evaluate asthma control perception [30]. 

Asthma treatment strategy and asthma severity were graded according to the GINA guidelines [2]. We consider chronic the use of oral corticosteroids (OCS) for at least 3 consecutive months in the last year. AEs were also retrieved and reported, and ≥2 AEs in the previous year identified frequent exacerbator phenotype [31,32]. Life-span history of serious AEs requiring emergency room access and hospitalization was reported. 

Allergen sensitization to perennial (house dust mites, cat and dog dander, and molds) and seasonal inhalants was detected by positive skin prick tests and/or serum allergen-specific IgE. Patients sensitized to ≥2 allergens were considered polysensitized.

T2_High_ inflammation occurred whether at least one condition among B-EOS > 300 cells/µL, F_E_NO ≥ 30 ppb or allergic sensitization existed [33]. We selected F_E_NO cut-off value ≥ 30 ppb, as previously found in our population [33]. Total serum IgE levels ≥ 100 kU/L were not considered per se as T2 inflammatory biomarkers, but this cut-off was used to categorize patients into two subgroups. This cut-off is derived from the median value of total serum IgE in our population, which is 102 kU/L, and from other cluster analyses that used 100kU/L as a discriminator for high and low IgE [13].

Patients were stratified according to both T2 inflammatory status and total serum IgE concentrations (IgE_High_, ≥100 kU/L).

### 2.3. Statistical Analysis 

In descriptive analyses, the ROUT method detected outliers to be excluded [34]. The deviation from normality was evaluated by the D’Agostino-Pearson test. Accordingly, one-way ANOVA (with Tukey post-hoc test) or Kruskal–Wallis H test (with Dunn post-hoc test) compared differences among groups. Chi-squared (χ^2^) tests (with Fisher’s exact post-hoc approach) [35] compared frequencies among groups.

In binomial and multinomial logistic regression models (LRMs), odds ratios (ORs) were calculated as exponentiation of the B-coefficient for each factor, which represents the odds of a unit change occurring in the independent variable in the analysis when all the others are kept constant. The 95% confidence interval (CI) was also reported. A *p*-value less than 0.05 was set as the significant cut-off. Variables for which a statistically significant difference resulted in comparing the groups were selected for further binomial logistic regression analysis. Variables giving significant odds or odds ≥ 2 were put into the multinomial LRM. The collinearity test for continuous variables was applied through inspection of correlation coefficients and accepted for variance inflation factors (VIF) values between 1 and 10.

Descriptive statistics and regression models were analyzed with IBM SPSS Statistics version 28 (IBM Corp., Armonk, NY, USA).

## 3. Results

### 3.1. Population General Characteristics

Descriptive statistics for the whole cohort of 547 asthmatic patients are reported in Table 1A–C. Most patients were female (62.7%), had late disease onset (76.4%), and had a long asthma history (24 ± 16 years); 28.4% were current or ex-smokers. Allergic sensitization was detected in 54.5% of patients, with the majority polysensitized (82.9%) (Table 1A). About 1/2 of patients (52.6%) could be classified into GINA Step 4 or 5 grades of severity, and 3/4 (75.5%) were partially or not controlled, while 15.9% had severe asthma treated with biological drugs (Table 1B). Upper airway inflammatory diseases resulted in the most common comorbidities, accounting for 64.0% and 35.4% of rhinitis and chronic rhinosinusitis (CRS), respectively (Table 1C). 

### 3.2. Patients’ Stratification into T2/IgE Phenotypes

**Figure 1:**
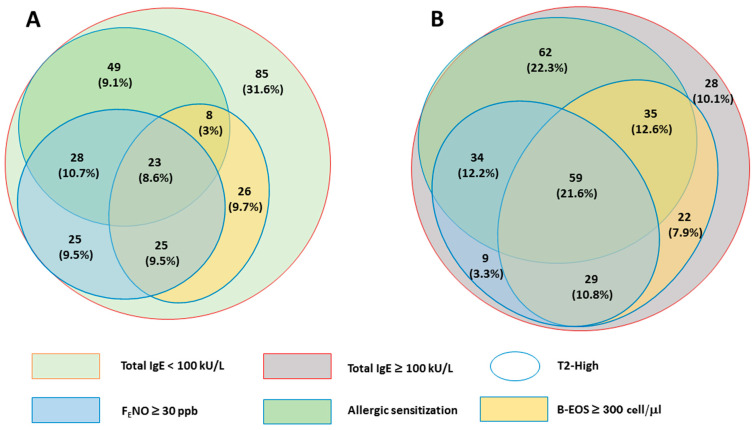
Area-proportional Euler diagrams of the type 2 biomarker positivity. (**A**) Population with Total Serum IgE < 100 kU/L; (**B**) Population with Total Serum IgE ≥ 100 kU/L.

Patients classifiable as T2_High_ due to the presence of one or more markers of T2 inflammation were 434 (79.3%). In detail, 298 (68.7%) had allergic sensitization, 232 (42.4%) had F_E_NO ≥ 30 ppb, and 227 (41.5%) reported B-EOS ≥ 300 cells/µL. The remaining 113 subjects (20.7%) were defined as T2_Low_ (Table 1A–C). Patients defined as IgE_High_ (IgE ≥ 100 kU/L) were 278 (50.8%), while 269 (49.2%) were considered IgE_Low_. The descriptive statistics of clinical and functional parameters among groups of patients stratified by a single biomarker were reported in Supplementary Table S1. The combination of T2 markers and total IgE status allowed us to classify four groups of patients: (1) T2_Low_ and IgE_Low_; (2) T2_Low_ and IgE_High_; (3) T2_High_ and IgE_Low_; and (4) T2_High_ and IgE_High_. The distribution of one to three positive T2 markers among IgE_Low_ (Figure 1A) and IgE_High_ (Figure 1B) patients is schematized by the Euler diagram.

In both diagrams, the blue line delimits the T2_High_ subgroup: the presence of allergic sensitization is represented in green; blood eosinophils ≥ 300 cells/µL are reported in yellow; F_E_NO ≥ 30 ppb is colored in azure; the presence of multiple T2 biomarkers is reported with the overlap of the respective biomarker ovals. The T2_Low_ subgroup, with (red) or without (teal) high total serum IgE levels, is represented outside the blue line.

### 3.3. Biomarker Expression

Both B-EOS and F_E_NO were significantly higher in the T2_High_ compared with the T2_Low_ groups, irrespective of their total serum IgE values (Table 1A and Table 2A). In detail, a mean T2_High_ B-EOS of 392 ± 493 cells/µL, 325 ± 299 cells/µL in IgE_Low_, and 443 ± 593 cells/µL in IgE_High_ resulted, respectively. Mean B-EOS were 156 ± 65 cells/µL (*p* < 0.001) in T2_Low_, 153 ± 65 cells/µL in IgE_Low_, and 161 ± 69 cells/µL in IgE_High_, respectively; mean T2_High_ F_E_NO was 44.0 ± 36.9 ppb, 43.0 ± 32.4 ppb in IgE_Low_, and 44.7 ± 40.1 ppb in IgE_High_; significantly increased compared with T2_Low_ F_E_NO of 14.7 ± 7.3 ppb (14.7 ± 7.3 ppb in IgE_Low_ and 13.6 ± 7.3 ppb in IgE_High_, respectively). Mean total IgE levels were significantly higher in the whole T2_High_ group (306.6 ± 572.3 kU/L) compared with the T2_Low_ group (131.2 ± 337.8 kU/L) but were comparable within both the IgE_High_ (499.6 ± 693.3 kU/L in T2_High_ and 437.6 ± 584.8 kU/L in T2_Low_, respectively) and the IgE_Low_ subgroups (44.3 ± 27.6 kU/L in T2_High_ and 30.2 ± 23.5 kU/L in T2_Low_). The T2_High_-IgE_High_ subjects reported higher allergen sensitization and prevalence of perennial allergens compared with the T2_High_-IgE_Low_ subjects (*p* < 0.001 and *p* < 0.05, respectively).

### 3.4. Characterization of T2High and T2Low Phenotypes

#### 3.4.1. Demographic Data, Lung Function, and Disease Presentation

T2_High_ and T2_Low_ patients significantly differed in terms of age, sex, and BMI, with T2_High_ patients being significantly younger, more frequently male, and thinner. T2_High_ had a shorter age of onset but did not differ in asthma duration (Table 1A). Significantly higher absolute FVC and FEV_1_ measures were detected in T2_High_ patients, as well as a lower RV/TLC ratio. Disease activity significantly differed between the two groups, with T2_Low_ subjects being worst controlled (*p* < 0.05) and more frequently in the GINA Step 4 level of treatment (*p* < 0.01) compared with T2_High_ patients, often needing LAMA add-on treatment, while T2_High_ patients were more frequently in GINA Step 5 (*p* < 0.05) (Table 1B).

#### 3.4.2. Comorbidities 

Rhinitis and nasal polyps were significantly more prevalent in T2_High_ compared with T2_Low_ patients (69.4% vs. 43.4%, *p* < 0.001, and 20.3% vs. 8.8%, *p* < 0.01, respectively). On the other hand, T2_Low_ patients reported higher rates of emphysema (21.2% vs. 9.9%, *p* < 0.01), obesity (31.9% vs. 20.3%, *p* < 0.05), diabetes mellitus (11.5% vs. 5.8%, *p* < 0.05), arterial hypertension (44.2% vs. 27.0%, *p* < 0.001), and acute myocardial infarction (8.0% vs. 3.5%, *p* < 0.05) (Table 1C). 

### 3.5. Characterization of T2 Phenotype According to Total Serum IgE Levels 

#### 3.5.1. Demographic Data, Lung Function, and Disease Presentation 

The two T2_High_ groups showed significant differences compared with the T2_Low_-IgE_Low_ group but less compared with the T2_Low_-IgE_High_ one (Table 2A,B).

Both T2_High_-IgE_High_ and T2_High_-IgE_low_ were characterized by a lower age (*p* < 0.001 and *p* < 0.05), earlier onset (*p* < 0.01 and *p* < 0.05), a higher proportion of male subjects (*p* < 0.001 and *p* < 0.05), and better lung function in terms of absolute FVC (*p* < 0.001 and *p* < 0.05) compared with the T2_Low_-IgE_Low_ group.

T2_High_-IgE_High_ had a lower BMI (*p* < 0.001), a higher AE rate (*p* < 0.05), and a significantly higher FEV_1_ (*p* < 0.001) compared with T2_Low_-IgE_Low_, while T2_High_-IgE_Low_ had a FRC% significantly lower than T2_Low_-IgE_Low_ (*p* < 0.05). 

Both T2_High_-IgE_High_ and T2_High_-IgE_Low_ were more often classified in GINA Step 5 and less in GINA Step 4 and used more frequently nasal corticosteroids (CS) compared with the T2_Low_-IgE_Low_ group (*p* < 0.001).

Some differences are also evident between the two T2_High_ groups. T2_High_-IgE_Low_ patients were more often female with a significantly higher BMI than the T2_High_-IgE_High_ patients (*p* < 0.05).

Within the two T2_Low_ groups, T2_Low_-IgE_High_ subjects showed a higher prevalence of male sex (*p* < 0.01), past smokers (*p* < 0.05), GINA Step 5 and Step 2 (*p* < 0.05), and lower FVC% (*p* < 0.05) compared with the T2_Low_-IgE_Low_ group (Table 1A,B). The T2_Low_-IgE_High_ group was also characterized by a higher rate of past smokers compared with all other groups (*p* < 0.05) and higher OCS and lower anti-leukotriene use compared with the T2_High_ subgroups. 

#### 3.5.2. Comorbidities 

The main comorbidities that differentiated T2_High_-IgE_High_ from T2_Low_-IgE_Low_ included a higher prevalence of rhinitis (*p* < 0.001) and chronic rhinosinusitis with nasal polyps (CRSwNP) (*p* < 0.01) and a lower occurrence of emphysema (*p* < 0.01), cardiovascular comorbidities (*p* < 0.05), GERD (*p* < 0.05), diabetes mellitus (*p* < 0.05), and obesity (*p* < 0.05). More frequently, rhinitis (*p* < 0.001) and CRSwNP (*p* < 0.01) were observed in T2_High_-IgE_Low_ patients compared with T2_Low_-IgE_Low_ patients (Table 2C). T2_Low_-IgE_High_ had a lower rate of rhinitis (*p* < 0.05) but a higher rate of OSAS (*p* < 0.01) and obesity (*p* < 0.05) compared with T2_High_-IgE_High_. Finally, within the T2_High_ subgroups, the presence of low IgE was associated with higher obesity (*p* < 0.05), arterial hypertension (*p* < 0.05), and anxiety/depression syndrome (*p* < 0.05).

### 3.6. Predictive Statistics (LRM)

When applying a binomial LRM to variables to predict T2 High versus T2 Low status, significantly higher odds were obtained for rhinitis (OR: 2.0), use of nasal CS (OR: 3.3), and GINA Step 5 (OR: 4.8), while lower risks were associated with emphysema (OR: 0.3) and poor ACT (OR: 0.3) (Table 3). The risks for obesity, diabetes, arterial hypertension, and acute myocardial infarction were not significant.

**Figure 2:**
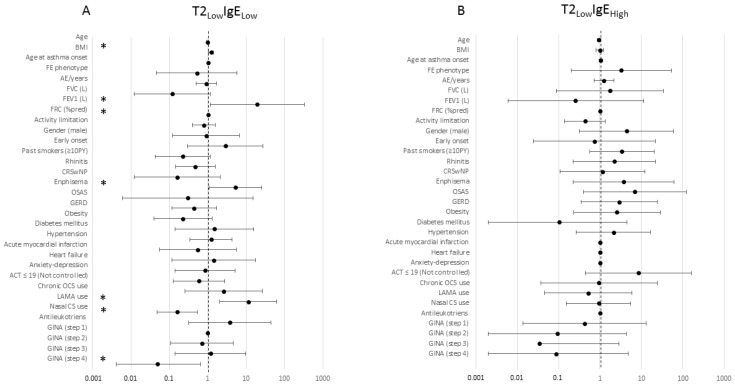

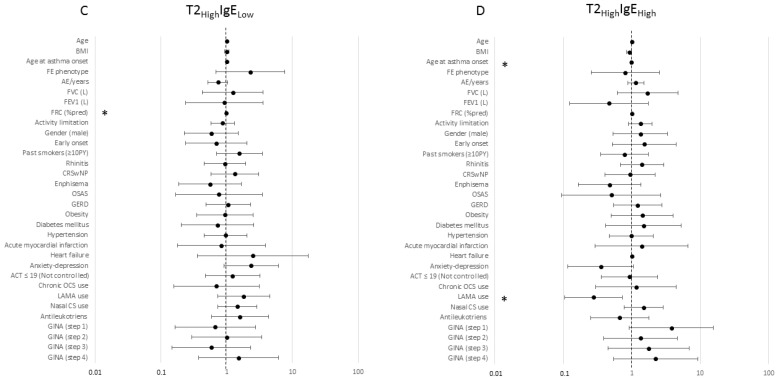
Forest diagrams representing odds from binomial LRM for patients stratified according to Total IgE and T2 inflammatory phenotypes. (**A**) T2_Low_-IgE_Low_ group; (**B**) T2_Low_-IgE_High_ group; (**C**) T2_High_-IgE_Low_ group; (**D**) T2_High_-IgE_High_ group. Odds for each single group are compared with the rest of the population (the union of the other three groups). X axis is expressed on a logarithmic scale. *: statistically significant.

When applying a binomial LRM for variables to predict the odds for each single subgroup compared with the rest of the population (Supplementary Table S2), major significant effects were found for factors identifying the T2_Low_-IgE_Low_ group, either positively (BMI OR: 1.2; FEV_1_ OR: 19.5; emphysema OR: 5.2; LAMA use OR: 11.3) or negatively (nasal CS use OR: 0.2). A forest graphic representation of binomial LMR is reported in Figure 2A–D.

After adjusting for colinearity, multinomial LRM was applied to predict the odds for variables associated with each subgroup compared with each of the others (Supplementary Table S3). The main factors associated with T2_Low_-IgE_Low_ compared with T2_High_-IgE_High_ were high BMI (OR: 1.1), emphysema (OR: 4.0), heart failure (OR: 8.0 × 10^8^), LAMA use (OR: 9.9), and poor control (OR: 3.6), while negative odds were found for rhinitis and nasal CS use compared with both the T2_High_ subgroups (*p* < 0.05).

Within the T2_Low_ groups, factors associated with high total serum IgE were the frequent exacerbator phenotype (OR: 7.9), rhinitis (OR: 8.5), and anxiety/depression (8.5 × 10^9^, *p* < 0.001).

**Figure 3:**
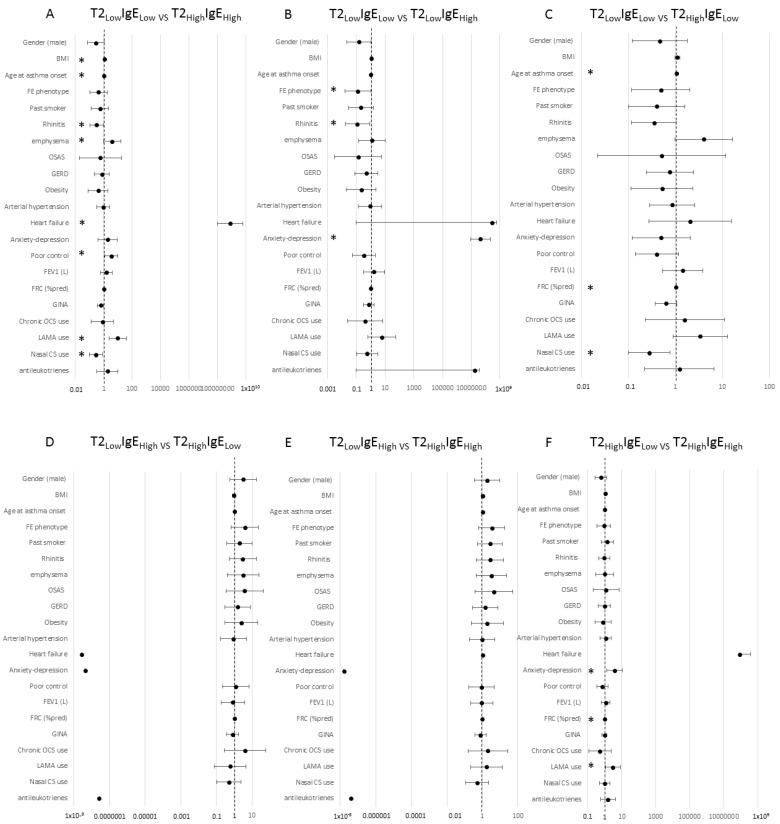
Forest Diagrams representing odds from Multinomial LRM for patients stratified according to Total IgE and T2 inflammatory phenotype. (**A**) T2_Low-_IgE_Low_ vs. T2_High-_IgE_High_; (**B**) T2_Low-_IgE_Low_ vs. T2_Low-_IgE_High_; (**C**) T2_Low-_IgE_Low_ vs. T2_High-_IgE_Low_; (**D**) T2_Low-_IgE_High_ vs. T2_High-_IgE_Low_; (**E**) T2_Low-_IgE_High_ vs. T2_High-_IgE_High_; (**F**) T2_High-_IgE_Low_ vs. T2_High-_IgE_High_. Odds for each single group are compared with each of the other three groups. X axis is expressed in a logarithmic scale. *: statistically significant.

Finally, the presence of anxiety/depression, use of LAMA, and lower FRC% could predict entry into the IgE_Low_ group within the T2_High_. A forest graphic representation of binomial LMR is reported in Figure 3A–F.

## 4. Discussion

Total serum IgE levels above a predefined cut-off have been widely used as a biomarker of type-2 inflammation in asthma. This paradigm derives from the expected association between high total serum IgE and the presence of allergen-specific IgE. High total serum IgE has also been found in “intrinsic” asthma, likewise characterized by eosinophilic airway inflammation [15]. However, many studies demonstrated IgE’s low predictivity of airway eosinophilia compared with B-EOS and F_E_NO [18], independently from the cut-off [23]. A systematic review and meta-analysis (including 942 patients) yielded for total IgE a sensitivity and specificity in detecting sputum eosinophils (>3%) of 0.64 and 0.71 [36]. The number of studies (n = 2) and the diagnostic accuracy (AUC range 0.62–0.64) of IgE dropped when S-EOS, bronchoalveolar lavage, or endobronchial biopsies were used as the reference standard. Moreover, a prospective trial reported that total serum IgE was less accurate in detecting S-EOS in allergic and obese asthmatics than in nonallergic and normal-weight asthmatics [18]. Therefore, the question of whether total serum IgE, either high or low, impacts the type-2_High_ and type-2_Low_ asthma phenotypes is still open.

The ISAR used a predefined total serum IgE cut-off of 75 kU/L or greater as a biomarker of type-2 severe asthma [9], while a Danish cohort study used ≥150 IU/mL [23,37]. In the current study, a total serum IgE cut-off ≥100 kU/L categorized patients, when combined with the expression of type-2 biomarkers, into four subgroups: T2_Low_ and IgE_Low_; T2_Low_ and IgE_High_; T2_High_ and IgE_Low_; and T2_High_ and IgE_High_. Their distribution revealed T2_High_-IgE_High_ as the most prevalent (45.7%), followed by T2_High_-IgE_Low_ (33.6%). Overall, T2_High_ patients accounted for 79.3% of the total population. These data were consistent with studies derived from moderate to severe unselected populations with T2_High_ prevalence ranging from 79.1% to 45.3% depending on whether allergy, B-EOS, or F_E_NO were chosen as selective criteria [22]. In our cohort, factors predictive of the T2_High_ phenotype were younger age, male sex, lower BMI, better lung function (FVC and FEV_1_) control, and a more frequent GINA Step 5 level of treatment. Moreover, rhinitis and nasal polyps were significantly more prevalent in T2_High_ patients compared with T2_Low_ patients. On the other hand, T2_Low_ subjects were worst controlled, more frequently in GINA Step 4 level of treatment, often needed LAMA as add-on treatment, and reported higher rates of comorbidities related to past smoking habits (emphysema), metabolic syndrome (obesity, diabetes mellitus), and cardiovascular disease (arterial hypertension, acute myocardial infarction). We applied a binomial logistic regression analysis to identify variables as predictors of T2_High_ versus T2_Low_ status, finding the highest odds for rhinitis, use of nasal CS, and GINA Step 5, and the lowest risks for emphysema and poor ACT. These findings are in line with previous reports [33]. It should be underlined that in our cohort, the high rate of GINA Step 4-5 patients, ex-smokers, and median long duration of disease (24 years) can justify the presence of airway obstruction in many patients independently from the T2 phenotype [33]. Both the T2_High_ and T2_Low_ groups may develop fixed airflow obstruction due to both eosinophilic and neutrophilic-derived airway remodeling [38].

The further sub-analysis detected two distinct populations within the T2_High_ and T2_Low_ phenotypes according to the serum IgE cut-off of 100 kU/L. This cut-off overlaps with the median value within our population (102 kU/L) and has been previously used both for cluster analysis in asthma populations [13] and type 2 biomarkers [33]. The T2_Low_-IgE_Low_ patients have no marker of type-2 inflammation. As expected, these subjects showed B-EOS and F_E_NO significantly lower and sharply below the thresholds of the T2_High_ groups. These patients had different clinical presentations and comorbidities than others. First, compared with the T2_High_ groups, they had more frequent cardiovascular comorbidities, known to be associated with an accelerated Th1/Th17 inflammatory milieu [39,40]. 

When applying a logistic regression analysis, the T2_Low_-IgE_Low_ group was positively identified by high BMI, low FEV_1_, emphysema, and LAMA use. Obesity was an important feature of this subset, composed mainly of older females with late-onset asthma, gastroesophageal reflux, and diabetes mellitus. A late-onset, obesity, high neutrophils, low eosinophils, and low IgE cluster in females was reported in a Taiwanese Adult Asthma Cohorts study [41]. Obesity, diabetes mellitus, and metabolic syndrome are diseases mediated by proinflammatory cytokines such as TNF-α, IL-1β, and IL-6 [42]. The SARP study showed, in a relevant proportion of ICS-naive mild-to-moderate asthmatics, a T2_Low_ asthma phenotype associated with frequent bronchial hyperresponsiveness (BHR) and a lower AE risk than subjects with T2_High_ disease [43]. In this context, different pathogenic processes may be involved, driven by IL-17, IL-6, and IL-23 or, alternatively, due to airway smooth muscle or neural dysfunction [5]. Moreover, different cluster analyses applied to moderate-to-severe asthma cohorts recurrently identified difficult-to-treat subsets of obese asthmatics, adult-onset, primarily affecting women, lacking T2 biomarkers, driven by oxidative stress or hormonal mechanisms. These patients have lower activity levels and frequent corticosteroid use due to symptom burden [44]. Exercise-induced airflow obstruction, ventilatory limitation, and lung mechanics alteration, unloading airway smooth muscle, and favoring fiber shortening during bronchoconstriction are involved in this phenotype [5]. We can suggest that our T2_Low_-IgE_Low_ patients collected both the mild and severe T2_Low_ asthmatics described in the above-mentioned cohorts. 

We here describe a small group of patients characterized by low T2 biomarkers and high total serum IgE levels (T2_Low_-IgE_High_). They differed from the T2_Low_-IgE_Low_ group in having a higher male and ex-smoker prevalence. Applying a logistic regression analysis model, the T2_Low_-IgE_High_ subjects showed significant odds of being AE-prone and suffering from rhinitis and anxiety/depression. In addition, sex (male), past smoking habits, OSAS, and obesity were also factors associated with high but not statistically significant odds for the T2_Low_-IgE_High_ group compared with the T2_Low_-IgE_Low_ group.

A similar phenotype has been described by a cluster analysis of inflammatory biomarker expression in the ISAR, in which cluster 4 was characterized by very high levels of total serum IgE (1932 kU/L), the longest duration of asthma, and a high BMI [9]. The Danish study above reported that patients with severe asthma (11% of the whole population) carried high total serum IgE levels as the only elevated T2 biomarker and were characterized by early onset disease, overweight, higher median pack years, and prednisone use compared with the other severe asthmatics [23]. What is interesting is that the median F_E_NO (14 ppb) and B-EOS (140 cell/µL) corresponded to those of our T2_Low_-IgE_High_ group. However, both the clusters above mentioned did not exclude patients with allergic sensitization, as we did. Therefore, we can assume that just a proportion of those populations corresponded to ours. Accordingly, cluster analysis from the U-BIOPRED network, based on sputum omics, reported that about half of the cluster T2 (obese, late-onset severe asthma, smoking history, and chronic airflow obstruction) were not allergic, but a sub-analysis of this group was not available [45]. Smoking and/or exposure to environmental pollutants have been associated with increased total serum IgE levels independently of allergen exposure. Cigarette smoking enhances the production of IgE antibodies, BHR, and leukotriene B4 (a potent stimulator of neutrophil chemotaxis) by leucocytes in elderly asthmatics, and in vitro, it appears to be associated with increased levels of IL-13 [46,47]. Finally, a further mechanism potentially involved in the IgE modulation is the expression of SNPs in FCεRII associated with increased IgE levels, steroid-refractory responses, and asthma exacerbations [48].

In our study, within the T2_High_ subgroups, the presence of low IgE was associated with higher obesity (*p* < 0.05), arterial hypertension (*p* < 0.05), and anxiety/depression syndrome (*p* < 0.05). This last feature, as well as the use of LAMA and a lower FRC%, could predict the entry of the IgE_Low_ group within the T2_High_. A cluster analysis study applied to allergic asthmatics described a similar cluster 1, gathering a high proportion of IgE-low patients (<100 kU/L), prevalently female, with mean F_E_NO levels of 44.3 ppb and mean B-EOS of 304.0/mL [13]. Within a severe asthma population, biomarker cluster analysis revealed cluster 2 of older exacerbating females with relatively low IgE [9]. Among the potential mechanisms influencing sex-related IgE levels, candidates are polymorphisms involving the IL21R promoter region, the IL4/IL13 pathway, and the CTLA-4 genes [10,49].

We can speculate that serum total IgE is a biomarker able to identify different and specific (sub)phenotypes in both T2 high and T2 low clusters. 

In a recent study, we described among a population of 503 mild to severe asthmatics that about 19.5% of patients could be classified as T2_Low_, triple “negative” for the expression of T2 biomarkers [33]. These patients had positive tests for high BMI, age onset, and smoking pack years. Moreover, these patients were more susceptible to cardiovascular and obesity-related comorbidities. We reported a median total serum IgE value of 44 UI/mL among these nonallergic T2_Low_ patients. Here, we extend the previous observation to a wider population, describing the two subgroups of T2_Low_-IgE_Low_ and T2_Low_-IgE_High_, which are different from a clinical point of view, as detailed above.

The strength of this study was the large cohort of included patients and the vast number of evaluated parameters. However, the study had some limitations. One limitation was the cross-sectional nature, such as the variability of biological measures along the natural history of a patient. Some data reported during a 5-year retrospective observation showed that 66.7% of participants had more than 50% alternations of IgE in two different measures [50]. However, fluctuations of IgE above or below the threshold of 100 kU/L we have chosen seemed to be limited and associated with a further small subgroup of patients. A longitudinal observation could reveal additional information, such as the influence of IgE levels on concomitant immune-mediated disease development. The elevated risk of cancer of any type in patients with ultra-low IgE levels is one of the most intriguing fields of research [51]. In addition, emotional and socioeconomic issues were not considered in this study. The high prevalence of severe asthma we reported is consistent with the real-life setting of a third-level asthma clinic, in which additional factors influence daily clinical practice [52].

## 5. Conclusions

This single-center cross-sectional study explored the role of total IgE levels among asthmatic patients classified into T2_High_ and T2_Low_ phenotypes according to the most routinely accepted measurable biomarkers. The role of a high level of IgE in T2_Low_ patients seems to be associated with a peculiar clinical phenotype, distinct not only from T2_High_ patients but also from patients with low total serum IgE and low T2 markers. Moreover, lower levels of total IgE in the context of a type-2 phenotype seem to confer a somehow different profile on the T2_High_ subgroup. Therefore, measuring total IgE could be useful in asthma workup and management as it may suggest clinically relevant information orienting towards forms of asthma with overlapping pathogenetic mechanisms involving IgE and other inflammatory pathways. According to a recent review [53], this study confirmed that different phenotype overlaps could appear in the natural history of asthma: for instance, an allergic T2 high phenotype could be associated with neutrophilic inflammation (Th17, Th1, Th3) and vice versa. A T2_low_ phenotype, such as in smokers or obese asthmatics, could move towards a high IgE response, generating novel phenotypes with pathognomonic clinical signs.

## Figures and Tables

**Table 1:** (**A**): Demographic, clinical, and bio-humoral parameters of asthmatic patients stratified according to T2 inflammatory phenotype. (**B**): Pulmonary function parameters, disease severity, and pharmacological treatments. (**C**): Comorbidities.

**(A)**				
**Characteristic**	**Overall**	**T2-Low**	**T2-High**	***p***
	**(N = 547)**	**(N = 113)**	**(N = 434)**	
**Age (years)**	59 ± 15 [60]	63 ± 15 [68]	57 ± 15 [59]	***
**Sex** (Male)	204 (37.3%)	33 (29.2%)	171 (39.4%)	*
**BMI** (N = 544)	27.2 ± 5.6 [26.2]	28.8 ± 5.3 [29]	26.7 ± 5.6 [25.9]	***
**Age at asthma onset**	35 ± 20 [35.0]	42 ± 20 [42]	33 ± 19 [34]	***
**Early onset**	129 (23.6%)	15 (13.3%)	114 (26.3%)	**
**Asthma duration (years)**	24 ± 16 [20]	22 ± 17 [17]	24 ± 16 [21]	
**Vitamin D (ng/mL)** (N = 427)	26.9 ± 13.5 [25]	27.5 ± 19.4 [23.2]	26.7 ± 11.7 [25.0]	
**AE/years** (N = 497)	1.12 ± 2.00 [0]	0.92 ± 1.07 [0]	1.17 ± 2.01 [0]	
**Frequent Exacerbator phenotype** (N = 497)	118 (23.7%)	19 (19.8%)	99 (24.7%)	
**Serious AE** (N = 513)	175 (34.1%)	36 (20.6%)	139 (33.7%)	
**Fibrinogen** (mg/dL) (N = 326)	341.5 ± 82.4 [331]	342.6 ± 74.4 [332]	341.2 ± 84.5 [331]	
**Current smokers** (≥10PY) (N = 544)	30 (5.5%)	6 (5.3%)	24 (5.5%)	
**Past smokers** (≥10PY) (N = 543)	125 (22.9%)	32 (28.3%)	93 (21.4%)	
**Leukocytes** (WBC cells/µL) (N = 542)	7293 ± 2147 [7000]	7220 ± 2122 [6775]	7312 ± 2156 [7070]	
**Lymphocytes** (cells/µL) (N = 396)	2319 ± 794 [2190]	2153 ± 575 [2060]	2363 ± 838 [2230]	*
**Monocytes** (cells/µL) (N = 366)	576 ± 214 [550]	553 ± 164 [530]	582 ± 225 [560]	
**Neutrophils** (cells/µL) (N = 482)	4039 ± 1533 [3775]	4209 ± 1738 [3890]	3995 ± 1475 [3760]	
**Basophils** (cells/µL) (N = 351)	47 ± 38 [40]	43 ± 32 [40]	47 ± 40 [40]	
**Eosinophils** (cells/µL) (N = 541)	344 ± 451 [240]	156 ± 65 [150]	392 ± 493 [300]	***
**F_E_NO** (ppb) (N = 498)	38.1 ± 35.2 [28]	14.5 ± 7.3 [13.7]	44.0 ± 36.9 [36.0]	***
**Total serum IgE** (kU/L)	270.3 ± 536.8 [102]	131.2 ± 337.8 [33.0]	306.6 ± 572.3 [126.5]	**
**Allergic sensitization**	298 (54.5%)	-	298 (68.7%)	
**Poly-sensitization**	247/298 (82.9%)	-	247/298 (82.9%)	
**Seasonal allergen sensitization**	246/298 (82.6%)	-	246/298 (82.6%)	
**Perennial allergen sensitization**	225/298 (75.5%)	-	225/298 (75.5%)	
**Moulds sensitization**	55/298 (18.5%)	-	55/298 (18.5%)	
**(B)**				
**Characteristic**	**Overall** **(N = 547)**	**T2-low** **(N = 113)**	**T2-high** **(N = 434)**	***p***
**FVC (%pred.)** (N = 536)	100.2 ± 19.2 [100.0]	99.6 ± 20.1 [98.5]	100.3 ± 18.7 [100.0]	
**FVC (L)** (N = 536)	3.11 ± 1.05 [2.98]	2.77 ± 0.97 [2.65]	3.19 ± 1.06 [3.12]	***
**FEV_1_ (%pred.)** (N = 540)	84.1 ± 21.4 [84.0]	84.5 ± 22.2 [83.0]	84.0 ± 22.2 [85.0]	
**FEV_1_ (L)** (N = 540)	2.14 ± 0.84 [2.07]	1.94 ± 0.82 [1.84]	2.19 ± 0.84 [2.12]	**
**FEV_1_/FVC (%)** (N = 536)	68.4 ± 12.4 [68.9]	68.8 ± 10.8 [69.5]	68.2 ± 12.8 [68.8]	
**∆-post-BD FVC (mL)** (N = 437)	230.8 ± 453.4 [160.0]	234.7 ± 484.2 [170]	229.7 ± 445.6 [160]	
**∆-post-BD FEV_1_ (mL)** (N = 469)	228.4 ± 243.8 [190.0]	203.9 ± 247.7 [160.0]	234.7 ± 242.7 [190.0]	
**RV (%pred.)** (N = 464)	127.3 ± 37.2 [122.0]	129.0 ± 33.4 [126.0]	126.7 ± 38.2 [122.0]	
**RV/TLC (%)** (N = 411)	45.3 ± 12.2 [44.9]	48.4 ± 11.6 [48.2]	44.5 ± 12.2 [43.9]	**
**TLC (%pred.)** (N = 446)	107.0 ± 15.0 [106.0]	106.7 ± 15.8 [108.0]	107.0 ± 14.8 [106.0]	
**FRC (%pred.)** (N = 270)	112.3 ± 25.1 [109.0]	117.4 ± 22.7 [118.0]	110.8 ± 25.7 [109.0]	
**FVC (%pred.)-post** (N = 438)	105.8 ± 19.3 [107.0]	105.6 ± 21.8 [106.5]	105.9 ± 18.6 [107.0]	
**FEV_1_ (%pred.)-post** (N = 465)	91.1 ± 21.6 [92.0]	90.3 ± 21.6 [90.0]	91.3 ± 21.6 [93.0]	
**FEV_1_/FVC (%)-post** (N = 436)	69.3 ± 11.1 [70.0]	69.0 ± 10.6 [69.0]	69.4 ± 11.2 [70.0]	
**SpO2 (%)** (N = 529)	96 ± 1 [97]	96 ± 2 [96]	97 ± 2 [97]	
**Heart Rate (bpm)** (N = 530)	78 ± 12 [77]	77 ± 12 [77]	78 ± 12 [78]	
**ACT**	20.1 ± 4.3 [21.0]	18.8 ± 4.7 [20.0]	20.5 ± 4.1 [22.0]	***
24–25 (Controlled)	134 (24.5%)	19 (16.8%)	115 (26.5%)	*
20–23 (Partially controlled)	213 (38.9%)	41 (36.3%)	172 (39.6%)	
≤19 (Not controlled)	200 (36.6%)	53 (46.9%)	147 (33.9%)	*
**Activity limitation**	4.0 ± 1.1 [4.0]	3.7 ± 1.2 [4.0]	4.1 ± 11 [5.0]	**
**Asthma severity grade**				
GINA Step 1	52 (9.5%)	12 (10.6%)	40 (9.2%)	
GINA Step 2	53 (9.7%)	7 (6.2%)	46 (10.6%)	
GINA Step 3	154 (28.2%)	35 (31.0%)	119 (27.4%)	
GINA Step 4	116 (21.2%)	34 (30.1%)	82 (18.9%)	**
GINA Step 5	172 (31.4%)	25 (22.1%)	147 (33.9%)	*
**BCM HFA dose (µg)**	364.1 ± 259.3 [300.0]	365.5 ± 263.1 [400.0]	363.7 ± 258.6 [300.0]	
**Chronic OCS** use	37 (6.8%)	12 (10.6%)	25 (5.8%)	
**LABA** use	447 (81.7%)	95 (84.1%)	352 (81.1%)	
**LAMA** use	94 (17.2%)	28 (24.8%)	66 (15.2%)	*
**Biologics ongoing**	39 (7.1%)	-	39 (7.1%)	
**Pre Biologics**	48 (8.8%)	-	48 (8.8%)	
**All Biologics**	87 (15.9%)	-	87 (15.9%)	
**Omalizumab**	37 (6.8%)	-	37 (6.8%)	
**Mepolizumab**	23 (4.2%)	-	23 (4.2%)	
**Benralizumab**	18 (3.3%)	-	18 (3.3%)	
**Dupilumab**	9 (1.6%)	-	9 (1.6%)	
**Nasal CS** use	325 (59.4%)	42 (37.2%)	283 (65.2%)	***
**Antileukotriene**	74 (13.5%)	7 (6.2%)	67 (15.4%)	*
**(C)**				
	**Overall**	**T2-low**	**T2-high**	***p***
	**(N = 547)**	**(N = 113)**	**(N = 434)**	
**Aspirin intolerance**	75 (13.7%)	13 (17.3%)	62 (14.3%)	
**Rhinitis**	350 (64.0%)	49 (43.4%)	301 (69.4%)	***
**CRSsNP**	96 (17.5%)	14 (12.4%)	82 (18.9%)	
**CRSwNP**	98 (17.9%)	10 (8.8%)	88 (20.3%)	**
**Bronchiectasis**	61 (11.2%)	13 (11.5%)	48 (11.1%)	
**Emphysema**	67 (12.2%)	24 (21.2%)	43 (9.9%)	**
**Pneumonia history**	71 (13.0%)	18 (15.9%)	52 (12.2%)	
**OSAS**	29 (5.3%)	8 (7.1%)	21 (4.8%)	
**GERD**	119 (21.8%)	32 (28.3%)	87 (20.0%)	
**Obesity**	124 (22.7%)	36 (31.9%)	88 (20.3%)	*
**Diabetes mellitus**	38 (6.9%)	13 (11.5%)	25 (5.8%)	*
**Arterial hypertension**	167 (30.5%)	50 (44.2%)	117 (27.0%)	***
**Acute myocardial infarction**	24 (4.4%)	9 (8.0%)	15 (3.5%)	*
**Heart failure**	8 (1.5%)	3 (2.7%)	5 (1.2%)	
**Arrhythmia**	38 (6.9%)	9 (8.0%)	29 (6.7%)	
**Anxiety-depression**	76 (13.9%)	15 (13.3%)	61 (14.1%)	
**Osteoporosis**	44 (8.0%)	10 (8.8%)	34 (7.8%)	
**Chronic Pain**	33 (6.0%)	9 (8.0%)	24 (5.5%)	
**Arthropathy**	48 (8.8%)	12 (10.6%)	36 (8.3%)	

The results are expressed as mean with standard deviation or as a number of subjects with percentage. * = *p* < 0.05, ** = *p* < 0.01, *** = *p* < 0.001; median is reported in brackets []; AE: asthma exacerbations; frequent exacerbator (≥2 exacerbation/year). The results are expressed as mean with standard deviation or as a number of subjects with percentage. * = *p* < 0.05, ** = *p* < 0.01, *** = *p* < 0.001; median is reported in brackets []; we consider chronic the use of OCS for at least 3 consecutive months in the last year. FVC: forced vital capacity; FEV_1_: forced expiratory capacity in the first second; BD: bronchodilator; RV: residual volume; TLC: total lung capacity; FRC: functional residual capacity; GINA: Global Initiative for Asthma; BCM HFA: beclomethasone extrafine formulation equivalent dose; LABA: Long-Acting Beta-Agonists; LAMA: long-acting muscarinic antagonist. The results are expressed as the number of subjects by percentage. * = *p* < 0.05, ** = *p* < 0.01, *** = *p* < 0.001; CRSsNP: chronic rhinosinusitis without polyps; CRSwNP: chronic rhinosinusitis with polyps; OSAS: obstructive sleep apnea syndrome; GERD: gastroesophageal reflux disease. Obesity = BMI ≥ 30.

**Table 2:** (**A**): Demographic, clinical, and bio-humoral parameters of asthmatic patients stratified according to serum Total Serum IgE and T2 inflammatory phenotype. (**B**): Pulmonary function parameters, disease severity, and pharmacological treatments. (**C**): Comorbidities.

**(A)**					
**Characteristics**	**Overall**	**T2-Low**		**T2-High**	
	**(N = 547)**	**IgE < 100 kU/L** **(N = 85)**	**IgE ≥ 100 kU/L** **(N = 28)**	**IgE < 100 kU/L** **(N = 184)**	**IgE ≥ 100 kU/L** **(N = 250)**
**Age (years)**	59 ± 15 [60]	65 ± 15 ^£££/#^ [68]	61 ± 17 [63.5]	59 ± 14 * [59.5]	56 ± 16 *** [58]
**Sex** (Male)	204 (37.3%)	19 (22.4%) ^£££/§§/#^	14 (50.0%) **	61 (33.2%) ^£/^*	110 (44.0%) ***^/#^
**BMI** (N = 544)	27.2 ± 5.6 [26.2]	28.8 ± 5.0 ^£££^ [28.6]	28.6 ± 6.1 [30]	27.6 ± 5.7 ^£^ [26.9]	26.1 ± 5.5 ***^/#^ [25.6]
**Age at asthma onset**	35 ± 20 [35]	43 ± 20 ^##/£££^ [45]	38 ± 18 [40]	34 ± 19 ** [35]	33 ± 19 *** [33]
**Early onset**	129 (23.6%)	10 (11.8%) ^#/££^	5 (17.9%)	46 (25.0%) *	68 (27.2%) **
**Asthma duration (years)**	24 ± 16 [20]	22 ± 17 [17]	22 ± 15 [18]	25 ± 18 [20]	23 ± 15 [21]
**Vitamin D (ng/mL)** (N = 427)	26.9 ± 13.5 [25]	26.6 ± 14.0 [24.3]	30.3 ± 31.7 [22.3]	26.5 ± 10.9 [25.0]	26.9 ± 12.2 [25.2]
**AE/years** (N = 497)	1.12 ± 2.00 [0]	0.65 ± 1.06 ^£^ [0]	1.90 ± 2.91 [1]	0.91 ± 1.66 [0]	1.36 ± 2.31 * [1]
**Frequent Exacerbator phenotype** (N = 497)	118 (23.7%)	12 (16.0%) ^£^	7 (33.3%)	37 (21.4%)	62 (27.2%) *
**Serious AE** (N = 513)	175 (34.1%)	27 (34.6%)	9 (40.9%)	55 (30.9%)	84 (35.7%)
**Fibrinogen** (mg/dL) (N = 326)	341.5 ± 82.4 [331.0]	347.5 ± 77.1 [333.0]	328.4 ± 65.9 [323.0]	341.1 ± 86.1 [334.0]	341.3 ± 83.6 [330.0]
**Current smokers** (≥10PY) (N = 544)	30 (5.5%)	6 (7.1%)	0 (0.0%)	12 (6.5%)	12 (4.8%)
**Past smokers** (≥10PY) (N = 543)	125 (22.9%)	20 (23.5%) ^§^	12 (42.9%) *^/#/£^	39 (21.2%) ^§^	54 (21.6%) ^§^
**Leukocytes** (cells/µL) (N = 542)	7293 ± 2147 [7000]	7153 ± 2016 [6730]	7417 ± 2441 [6855]	7079 ± 1872 [6670]	7483 ± 2332 [7220]
**Lymphocytes** (cells/µL) (N = 396)	2319 ± 794 [2190]	2156 ± 533 [2110]	2144 ± 715 [1930]	2313 ± 797 [2155]	2398 ± 867 [2300]
**Monocytes** (cells/µL) (N = 366)	576 ± 214 [550]	560 ± 146 [540]	529 ± 218 [490]	550 ± 211 [520]	605 ± 232 [580]
**Neutrophils** (cells/µL) (N = 482)	4039 ± 1533 [3775]	4153 ± 1729 [3820]	4382 ± 1790 [3935]	3843 ± 1342 [3650]	4106 ± 1559 [3790]
**Basophils** (cells/µL) (N = 351)	47 ± 38 [40]	40 ± 33 [40]	33 ± 28 [30]	50 ± 33 [50]	45 ± 44 [40]
**Eosinophils** (cells/µL) (N = 541)	344 ± 451 [240]	153 ± 65 ^£££/#^ [150]	161 ± 69 ^£^ [160]	325 ± 299 ^*/£^ [245]	443 ± 593 ***^/§/#^ [330]
**F_E_NO** (ppb) (N = 498)	38.1 ± 35.2 [28]	14.7 ± 7.3 ^£££/###^ [14.0]	13.6 ± 7.3 ^£££/###^ [13.0]	43.0 ± 32.4 ***^/§§§^ [36.5]	44.7 ± 40.1 ***^/§§§^ [35.0]
**Total serum IgE** (kU/L)	270.3 ± 536.8 [102]	30.2 ± 23.5 ^£££/§§§^ [26.2]	437.6 ± 584.8 ***^/###^ [200.5]	44.3 ± 27.6 ^£££/§§§^ [40.9]	499.6 ± 693.3 ***^/###^ [271.5]
**Allergic sensitization**	298 (54.5%)	0 (0.0%)	0 (0.0%)	108/184 (58.7%) ^£££^	190/250 (76.0%) ^###^
**Poly-sensitization**	247/298 (82.9%)	-	-	86/108 (79.6%)	161/190 (84.7%)
**Seasonal allergen sensitization**	246/298 (82.6%)	-	-	87/108 (80.6%)	159/190 (83.7%)
**Perennial allergen sensitization**	225/298 (75.5%)	-	-	73/108 (67.6%) ^£^	152/190 (80.0%) ^#^
**Moulds sensitization**	55/298 (18.5%)			17/108 (15.7%)	38/190 (20.0%)
**(B)**					
**Characteristics**	**Overall**	**T2-low**		**T2-high**	
	**(N = 547)**	**IgE < 100 kU/L** **(N = 85)**	**IgE ≥ 100 kU/L** **(N = 28)**	**IgE < 100 kU/L** **(N = 184)**	**IgE ≥ 100 kU/L** **(N = 250)**
**FVC (%pred.) (N = 536)**	100.2 ± 19.2 [100.0]	102.3 ± 19.2 ^§^ [100.5]	91.4 ± 23.9 * [96.0]	100.5 ± 19.9 [100.5]	100.1 ± 17.8 [100.0]
**FVC (L)** (N = 536)	3.11 ± 1.05 [2.98]	2.72 ± 0.89 ^£££/#^ [2.65]	2.93 ± 1.17 [2.70]	3.10 ± 1.02 * [2.98]	3.26 ± 01.08 *** [3.20]
**FEV_1_ (%pred.)** (N = 540)	84.1 ± 21.4 [84.0]	86.3 ± 20.5 [87.0]	79.1 ± 26.5 [80.5]	85.1 ± 20.9 [86.0]	83.2 ± 21.4 [83.0]
**FEV_1_ (L)** (N = 540)	2.14 ± 0.84 [2.07]	1.88 ± 0.71 ^££^ [1.84]	2.08 ± 1.06 [1.74]	2.15 ± 0.78 [2.11]	2.23 ± 0.88 ** [2.16]
**FEV_1/_FVC (%)** (N = 536)	68.4 ± 12.4 [68.9]	68.6 ± 10.3 [69.8]	69.2 ± 12.2 [67.5]	68.9 ± 10.0 [69.6]	67.7 ± 14.6 [67.9]
**D-post-BD FVC (mL)** (N = 437)	230.8 ± 453.4 [160.0]	247.0 ± 542.8 [1.70]	192.8 ± 182.4 [1.70]	221.8 ± 393.3 [1.60]	235.2 ± 479.2 [1.60]
**D-post-BD FEV_1_ (mL)** (N = 469)	228.4 ± 243.8 [190.0]	201.1 ± 272.2 [1.45]	213.0 ± 147.6 [2.00]	224.4 ± 254.7 [1.90]	241.9 ± 234.4 [1.90]
**RV (%pred.)** (N = 464)	127.3 ± 37.2 [122.0]	127.3 ± 33.2 [122.0]	134.9 ± 33.1 [137.5]	122.9 ± 31.2 [120.0]	129.9 ± 42.6 [123.5]
**RV/TLC (%)** (N = 411)	45.3 ± 12.2 [44.9]	48.0 ± 11.1 [48.3]	49.6 ± 13.1 [46.8]	45.1 ± 12.1 [43.6]	44.1 ± 12.3 [44.8]
**TLC (%pred.)** (N = 446)	107.0 ± 15.0 [106.0]	107.4 ± 16.2 [108.0]	105.2 ± 14.3 [107.0]	105.9 ± 14.5 [106.0]	107.8 ± 14.9 [106.0]
**FRC (%pred.)** (N = 270)	112.3 ± 25.1 [109.0]	117.4 ± 25.0 ^#^ [117.0]	117.2 ± 22.6 [121.0]	105.5 ± 22.8 ^£/^* [105.0]	114.6 ± 26.9 ^#^ [111.5]
**FVC (%pred.)-post** (N = 438)	105.8 ± 19.3 [107.0]	107.7 ± 17.1 [107.0]	98.5 ± 30.8 [105.0]	106.5 ± 19.5 [105.0]	105.5 ± 17.9 [107.0]
**FEV_1_ (%pred.)-post** (N = 465)	91.1 ± 21.6 [92.0	92.3 ± 19.9 [91.0]	81.7 ± 24.8 [81.0]	92.3 ± 21.6 [95.0]	90.6 ± 21.6 [92.0]
**FEV_1/_FVC (%)-post** (N = 436)	69.3 ± 11.1 [70.0]	69.2 ± 10.5 [70.0]	68.2 ± 11.1 [66.0]	70.4 ± 10.0 [71.0]	68.6 ± 11.9 [69.5]
**SpO2 (%)** (N = 529)	96 ± 1 [97.0]	96 ± 1 [96]	97 ± 2 [96]	97 ± 1 [97]	97 ± 2 [97]
**Heart Rate (bpm)** (N = 530)	78 ± 12 [77.0]	76 ± 12 [76]	79 ± 12 [78]	77 ± 12 [76]	79 ± 12 [79]
**ACT**	20.1 ± 4.3 [21.0]	19.2 ± 4.3 [21.0]	17.7 ± 5.9 [19.5]	20.6 ± 4.1 [22.0]	20.4 ± 4.1 [21.5]
24–25 (Controlled)	134 (24.5%)	14 (16.5%)	5 (17.9%)	50 (27.2%)	65 (26.0%)
20–23 (Partially controlled)	213 (38.9%)	32 (37.6%)	9 (32.1%)	74 (40.2%)	98 (39.2%)
≤19 (Not controlled)	200 (36.6%)	39 (45.9%) ^#^	14 (50.0%)	60 (32.6%) *	87 (34.8%)
**Activity limitation**	4.0 ± 1.1 [4.0]	3.8 ± 1.1 [4.0]	3.4 ± 1.4 ^#/£^ [4.0]	4.1 ± 1.1 ^§^ [5.0]	4.1 ± 1.0 ^§^ [4.0]
**Asthma severity grade**					
GINA Step 1	52 (9.5%)	9 (10.6%)	3 (10.7%)	19 (10.3%)	21 (8.4%)
GINA Step 2	53 (9.7%)	3 (3.5%) ^§/#/£^	4 (14.3%) *	19 (10.3%) *	27 (10.8%) *
GINA Step 3	154 (28.2%)	31 (36.5%) ^§/£^	4 (14.3%) *	57 (31.0%)	62 (24.8%) *
GINA Step 4	116 (21.2%)	27 (31.8%) ^#/£^	7 (25.0%)	34 (18.5%) *	48 (19.2%) *
GINA Step 5	172 (31.4%)	15 (17.6%) ^#/§/£££^	10 (35.7%) *	55 (29.9%) *	92 (36.8%) ***
**BCM HFA dose (µg)**	364.1 ± 259.3 [300.0]	338.8 ± 242.6 [300]	446.4 ± 308.5 [400]	346.4 ± 245.6 [200]	376.7 ± 267.6 [300]
**Chronic OCS** use	37 (6.8%)	7 (8.2%)	5 (17.9%) ^##/£^	6 (3.3%) ^§§^	19 (7.6%) ^§^
**LABA** use	447 (81.7%)	73 (85.9%)	22 (78.6%)	145 (78.8%)	207 (82.8%)
**LAMA** use	94 (17.2%)	21 (24.7%) ^£^	7 (25.0%)	29 (15.8%)	37 (14.8%) *
**Biologics ongoing**	39 (7.1%)	0 (0.0%)	0 (0.0%)	9 (4.9%)	30 (12%)
**Pre Biologics**	48 (8.8%)	0 (0.0%)	0 (0.0%)	16 (8.7%)	32 (12.8)
**All Biologics**	87 (15.9%)	0 (0.0%)	0 (0.0%)	25 (13.6%) ^££^	62 (24.8%) ^##^
**Omalizumab**	37 (6.8%)	0 (0.0%)	0 (0.0%)	10 (5.4%) ^£^	27 (10.8%) ^#^
**Mepolizumab**	23 (4.2%)	0 (0.0%)	0 (0.0%)	5 (2.7%) ^£^	18 (7.2%) ^#^
**Benralizumab**	18 (3.3%)	0 (0.0%)	0 (0.0%)	7 (3.8%)	11 (4.4%)
**Dupilumab**	9 (1.6%)	0 (0.0%)	0 (0.0%)	3 (1.6%)	6 (2.4%)
**Nasal CS** use	325 (59.4%)	28 (32.9%) ^###/£££^	14 (50.0%)	117 (63.6%) ***	166 (66.4%) ***
**Antileukotriene**	74 (13.5%)	7 (8.2%) ^£^	0 (0.0%) ^££/#^	25 (13.6%) ^§^	42 (16.8%) ^§§/^*
**(C)**					
	**Overall**	**T2-low**		**T2-high**	
	**(N = 547)**	**IgE < 100 kU/L** **(N = 85)**	**IgE ≥ 100 kU/L** **(N = 28)**	**IgE < 100 kU/L** **(N = 184)**	**IgE ≥ 100 kU/L** **(N = 250)**
**Aspirin intolerance**	75 (13.7%)	8 (9.4%)	5 (17.9%)	24 (13.0%)	38 (15.2%)
**Rhinitis**	350 (64.0%)	34 (40.0%) ^###/£££^	15 (53.6%) ^£^	120 (65.2%) ***	181 (72.4%) ***^/§^
**CRSsNP**	96 (17.5%)	11 (12.9%)	3 (10.7%)	34 (18.5%)	48 (19.2%)
**CRSwNP**	98 (17.9%)	6 (7.1%) ^#/££^	4 (14.3%)	33 (17.9%) *	55 (22.0%) **
**Bronchiectasis**	61 (11.2%)	9 (10.6%)	4 (14.3%)	19 (10.3%)	29 (11.6%)
**Emphysema**	67 (12.2%)	19 (22.4%) ^##/££^	5 (17.9%)	16 (8.7%) **	27 (10.8%) **
**Pneumonia history**	71 (13.0%)	14 (16.5%)	4 (14.3%)	28 (15.2%)	25 (10%)
**OSAS**	29 (5.3%)	4 (4.7%)	4 (14.3%) ^£^	10 (5.4%) ^§^	11 (4.4%) ^§^
**GERD**	119 (21.8%)	23 (27.1%) ^£^	9 (31.1%)	44 (23.9%)	43 (17.2%) *
**Obesity**	124 (22.7%)	25 (29.4%) ^£^	11 (39.3%) ^££^	46 (25%) ^£^	42 (16.8%) *^/#/§§^
**Diabetes mellitus**	38 (6.9%)	11 (12.9%) ^£^	2 (7.1%)	13 (7.1%)	12 (4.8%) *
**Arterial Hypertension**	167 (30.5%)	40 (47.1%) ^#/£££^	10 (35.7%)	61 (33.2%) *^/£^	56 (22.4%) ***^/#^
**Acute myocardial infarction**	24 (4.4%)	8 (9.4%) ^£^	1 (3.6%)	7 (3.8%)	8 (3.2%) *
**Heart failure**	8 (1.5%)	3 (3.5%) ^£^	0 (0.0%)	5 (2.7%) ^£^	0 (0.0%) *^/#^
**Arrhythmia**	38 (6.9%)	7 (8.2%)	2 (7.1%)	15 (8.2%)	14 (5.6%)
**Anxiety-depression**	76 (13.9%)	13 (15.6%)	2 (7.1%)	35 (19.0%) ^£^	26 (10.4%) ^#^
**Osteoporosis**	44 (8.0%)	8 (9.4%)	2 (7.1%)	13 (7.1%)	21 (8.4%)
**Chronic Pain**	33 (6.0%)	8 (9.4%)	1 (3.6%)	13 (7.1%)	11 (4.4%)
**Arthropathy**	48 (8.8%)	11 (12.9%)	1 (3.6%)	19 (10.3%)	17 (6.8%)

The results are expressed as mean with standard deviation or as a number of subjects with percentage. * = *p* < 0.05, ** = *p* < 0.01, *** = *p* < 0.001 vs. IgE_Low_-T2_Low_; # = *p* < 0.05, ## = *p* < 0.01, ### = *p* < 0.001 vs. IgE_Low_-T2_High_; § = *p* < 0.05, §§ = *p* < 0.01, §§§ = *p* < 0.001 vs. IgE_High_-T2_Low_; £ = *p* < 0.05, ££ = *p* < 0.01, £££ = *p* < 0.001 vs. IgE_High_-T2_High_; median is reported in brackets []; AE: asthma exacerbations; Frequent exacerbator (≥2 exacerbation/year); The results are expressed as mean with standard deviation or as a number of subjects with percentage. * = *p* < 0.05, ** = *p* < 0.01, *** = *p* < 0.001 vs. IgE_Low_-T2_Low_; # = *p* < 0.05, ## = *p* < 0.01, ### = *p* < 0.001 vs. IgE_Low_-T2_High_; § = *p* < 0.05, §§ = *p* < 0.01, §§§ = *p* < 0.001 vs. IgE_High_-T2_Low_; £ = *p* < 0.05, ££ = *p* < 0.01, £££ = *p* < 0.001 vs. IgE_High_-T2_High_; median is reported in brackets []; we consider as chronic the use of OCS for at least 3 consecutive months in the last year. FVC: forced vital capacity; FEV_1_: forced expiratory capacity in the first second; BD: bronchodilator; RV: residual volume; TLC: total lung capacity; FRC: functional residual capacity; GINA: Global Initiative for Asthma; BCM HFA: beclomethasone extrafine formulation equivalent dose; LABA: Long-Acting Beta-Agonists; LAMA: long-acting muscarinic antagonist. The results are expressed as the number of subjects by percentage.* = *p* < 0.05, ** = *p* < 0.01, *** = *p* < 0.001 vs. IgE_Low_-T2_Low_; # = *p* < 0.05, ## = *p* < 0.01, ### = *p* < 0.001 vs. IgE_Low_-T2_High_; § = *p* < 0.05, §§ = *p* < 0.01, §§§ = *p* < 0.001 vs. IgE_High_-T2_Low_; £ = *p* < 0.05, ££ = *p* < 0.01, £££ = *p* < 0.001 vs. IgE_High_-T2_High_; CRSsNP: chronic rhinosinusitis without polyps; CRSwNP: chronic rhinosinusitis with polyps; OSAS: obstructive sleep apnoea syndrome; GERD: gastroesophageal reflux disease. Obesity = BMI ≥ 30.

**Table 3:** Binomial logistic regression analysis of variables for asthmatic patients stratified according to T2 inflammatory phenotype.

Characteristic	T2_High_ vs. T2_Low_		
	OR	LB	UB
**Age**	0.997	0.957	1.038
**BMI**	0.947	0.869	1.033
**Age at asthma onset**	0.983	0.958	1.009
**FVC (L)**	2.731	0.825	9.041
**FEV_1_ (L)**	0.265	0.045	1.572
**RV/TLC**	0.190	0.000	179.421
**Lymphocytes**	1.000	0.000	1.001
**Sex (male)**	1.107	0.409	2.996
**Early onset**	0.791	0.215	2.906
**Rhinitis**	2.017 *	0.990	4.112
**CRSwNP**	0.629	0.213	1.854
**Emphysema**	0.268 *	0.093	0.769
**Obesity**	1.115	0.392	3.174
**Diabetes mellitus**	1.381	0.371	5.138
**Arterial hypertension**	1.118	0.507	2.468
**Acute myocardial infarction**	0.663	0.152	2.890
**ACT**	*		
**ACT ≤ 19 (Not controlled)**	0.312 *	0.087	1.124
**ACT ≥ 24 (well controlled)**	0.309	0.055	1.720
**LAMA use**	0.550	0.198	1.526
**Nasal CS use**	3.345 ***	1.637	6.835
**Antileukotriens**	5.546	0.913	33.673
**Asthma severity grade**	*		
**GINA (Step 1)**	2.179	0.395	12.004
**GINA (Step 2)**	1.113	0.278	4.449
**GINA (Step 3)**	0.899	0.198	4.081
**GINA (Step 4)**	4.375	0.874	21.904
**GINA (Step 5)**	4.759 **	1.645	13.768

* = *p* < 0.05, ** = *p* < 0.01, *** = *p* < 0.001. Bold font highlights statistically significant results. LB: lower bound; UB: upper bound; FVC: forced vital capacity; FEV_1_: forced expiratory capacity in the first second; RV: residual volume; TLC: total lung capacity; CRSwNP: Chronic Rhinosinusitis with Nasal Polyps; LAMA: long-acting muscarinic antagonist; GINA: Global Initiative for Asthma.

## Data Availability

Data are available on request from the authors.

## Supplementary Materials

The following supporting information can be downloaded at: https://www.mdpi.com/article/10.3390/jcm12175447/s1. Table S1: Descriptive statistics of clinical, bio-humoral and functional parameters among patients stratified by single biomarker cut-offs; Table S2: Binomial LRM of variables for asthmatic patients stratified according to serum Total IgE and T2 inflammatory phenotype; Table S3: Multinomial LRM of variables comparing each group of patients stratified according to serum Total IgE and T2 inflammatory phenotype.
