# Supplementary material for: Reliability of Total Serum IgE Levels to Define Type 2 High and Low Asthma Phenotypes
Source: J Clin Med. 2023 Aug 22;12(17):5447. doi: 10.3390/jcm12175447 (PMC10488214; doi:10.3390/jcm12175447)
Supplement: Supplementary file 1 [file jcm-12-05447-s001.zip › jcm-2533113-supplementary.pdf]

Supplementary Table S1: Descriptive statistics of clinical, bio-humoral and functional parameters among patients stratified by single biomarker cut-offs

Characteristic	*IgE ≥100KU/L (N=278)*	*IgE <100KU/L (N=269)*	*FENO≥30ppb (N=232)*	*FENO<30ppb (N=266)*	*B-EOS≥300cells/µL* (N=227)	*B-EOS<300cells/µL (N=314)*
**Age (years)**	56±16	61±14***	57±15	60±15*	59±15	59±16
**Gender** (*Male*)	124 (44.6%)	80 (29.7%)****	92 (39.7%)	99 (37.2%)	89 (39.2%)	114 (36.3%)
**BMI**	26.4±5.6	28.0±5.5***	26.1±5.5	27.9±5.7***	26.9±5.8	27.4±5.5
**Age at asthma onset**	33±19	37±20*	34±19	35±20	36±18	34±21
**Early onset**	73 (26.3%)	56 (20.8%)	50 (21.6%)	66 (24.8%)	40 (17.6%)	87 (27.7%)**
**Asthma duration (years)**	23±15	24±17	23±16	24±16	22±16	25±17
**Vitamin D (ng/mL)**	27.2±14.9	26.5±11.9	26.2±9.9	27.6±16.0	26.0±10.4	27.5±15.4
**AE/years**	1.40±2.37	0.83±1.50***	1.33±2.35	0.89±1.56*	1.26±1.29	1.02±1.77
**FE phenotype**	69 (27.7%)	49 (19.8%)*	57 (26.6%)	49 (20.4%)	53 (24.9%)	65 (23.1%)
**Serious AE**	93 (36.2%)	82 (32.0%)	79 (35.6%)	82 (50.9%)	76 (35.7%)	99 (33.6%)
**Fibrinogen** (mg/dL)	340.0±81.9	343.0±83.2	340.1±81.5	344.6±82.8	347.1±84.0	336.6±81.2
**Current smokers** (≥10PY)	12 (4.3%)	18 (6.7%)	11 (4.7%)	18 (6.8%)	12 (5.3%)	18 (5.7%)
**Past smokers** (≥10PY)	66 (23.7%)	59 (21.9%)	43 (18.5%)	70 (26.3%)*	50 (22.0%)	75 (23.9%)
**Leukocytes** (cells/µL)	7476±2339	7102±1914*	7340±2252	7213±2045	7779±2259	6944±1999****
**Lymphocytes** (cells/µL)	2374±855	2261±723	2350±781	2315±808	2483±889	2200±697****
**Monocytes** (cells/µL)	594±231	553±192*	576±199	560±191	629±234	538±189****
**Neutrophils** (cells/µL)	4133±1581	3941±1479	3968±1458	4043±1533	4068±1474	4016±1578
**Basophils** (cells/µL)	44±43	49±33	46±34	49±42	56±45	40±31****
**Eosinophils** (cells/µL)	414±569	272±264****	456±622	253±213****	-	-

**F_ENO** (ppb)	41.8±39.2	34.4±30.3*	-	-	48.1±39.1	31.1±30.3****
**Total serum IgE** (kU/L)	-	-	344.6±709.3	207.2±324.4**	364.9±700.1	203.6±369.3***
**Allergic sensitization**	190 (63.8%)	108 (40.1%)****	144 (62.1%)	132 (49.6%)**	125 (55.1%)	170 (54.1%)
**FVC(%pred.)**	99.2±18.7	101.1±19.6	100.7±19.8	100.1±18.3	99.6±18.6	100.3±19.5
**FEV_1(%pred.)**	82.8±21.9	85.5±20.8	84.4±21.6	84.0±20.9	82.7±20.5	84.6±21.8
**FEV_1/FVC (%)**	67.9±14.3	68.8±10.1	68.7±13.5	68.0±11.7	67.4±13.8	68.8±11.2
**RV (%pred.)**	130.4±41.9	124.4±32.0	128.3±39.8	126.4±35.1	128.1±38.5	127.5±35.6
**ACT**	20.1±4.4	20.2±4.2	20.3±4.1	20.3±4.2	20.4±4.1	19.9±4.4
**BCM HFA dose (µg)**	383.7±272.2	343.8±244.2	382.2±263.9	331.9±242.0*	394.7±261.2	345.2±254.9**
**Chronic OCS** use	24 (8.6%)	13 (4.8%)	15 (6.5%)	16 (6.0%)	17 (7.5%)	20 (6.4%)
**LABA** use	229 (82.4%)	218 (81.0%)	187 (80.6%)	216 (81.2%)	195 (85.9%)	249 (79.3%)
**LAMA** use	44 (15.8%)	50 (18.6%)	34 (14.7%)	52 (19.5%)	36 (15.9%)	58 (18.5%)
**Biologics ongoing**	30 (10.8%)	9 (3.3%)	20 (8.6%)	17 (6.4%)	23 (10.1%)	16 (5.1%)
**Pre Biologics**	32 (11.5%)	16 (5.9%)	29 (12.5%)	16 (6.0%)	36 (15.9%)	12 (3.8%)

The results are expressed as mean with standard deviation or as a number of subjects with percentage. *=$p<0.05$; **=$p<0.01$; ***=$p<0.001$; ****=$p<0.0001$.

Supplementary Table S2: Binomial LRM of variables for asthmatic patients stratified according to serum Total IgE and T2 inflammatory phenotype

Characteristic	*$T2_{Low}$-IgE_{Low}*			*$T2_{Low}$-IgE_{High}*			*$T2_{High}$-IgE_{Low}*			*$T2_{High}$-IgE_{High}*		
	OR	LB	UB	OR	LB	UB	OR	LB	UB	OR	LB	UB
**Age**	0.990	0.932	1.051	0.948	0.857	1.049	1.007	0.975	1.039	1.005	0.974	1.036
**BMI**	**1.222**[*]	1.047	1.427	0.987	0.798	1.222	1.008	0.930	1.092	0.920	0.843	1.004
**Age at asthma onset**	1.036	0.991	1.083	1.037	0.961	1.119	1.004	0.978	1.030	**0.975***	0.950	1.000
**FE phenotype**	0.515	0.046	5.724	3.287	0.203	53.112	2.304	0.688	7.715	0.805	0.254	2.546
**AE/years**	0.922	0.501	1.698	1.248	0.714	2.181	0.738	0.524	1.038	1.148	0.873	1.510
**FVC (L)**	0.118	0.012	1.163	1.740	0.090	33.791	1.249	0.433	3.605	1.702	0.610	4.747
**FEV_1 (L)**	**19.528***	1.165	327.304	0.255	0.006	11.441	0.922	0.238	3.570	0.463	0.124	1.729
**FRC (%pred)**	**1.040***	1.007	1.075	1.018	0.980	1.057	**0.978***	0.962	0.994	1.010	0.994	1.026
**Activity limitation**	0.789	0.391	1.592	0.432	0.140	1.333	0.874	0.580	1.318	1.333	0.893	1.989
**Gender (male)**	0.900	0.121	6.702	4.339	0.318	59.183	0.591	0.230	1.520	1.333	0.531	3.344
**Early onset**	2.854	0.298	27.328	0.729.	0.025	21.687	0.696	0.238	2.029	1.523	0.522	4.443
**Past smokers (≥10PY)**	0.226	0.043	1.185	3.403	0.571	20.303	1.569	0.703	3.501	0.783	0.354	1.733
**Rhinitis**	0.476	0.147	1.542	2.211	0.221	22.124	0.947	0.461	1.946	1.416	0.682	2.939
**CRSwNP**	0.158	0.012	2.100	1.162	0.109	12.334	1.343	0.581	3.103	0.940	0.403	2.196

**Emphysema**	**5.244***	1.088	25.271	3.747	0.228	61.479	0.563	0.187	1.689	0.474	0.166	1.355
**OSAS**	0.303	0.006	15.449	7.007	0.409	120.083	0.766	0.167	3.506	0.503	0.095	2.661
**GERD**	0.436	0.114	1.658	2.909	0.353	24.001	1.061	0.486	2.314	1.218	0.541	2.745
**Obesity**	0.223	0.039	1.286	2.559	0.230	28.445	0.943	0.353	2.516	1.419	0.499	4.036
**Diabetes mellitus**	1.493	0.141	15.840	0.105	0.002	4.495	0.731	0.206	2.588	1.483	0.413	5.322
**Arterial hypertension**	1.222	0.342	4.361	2.116	0.271	16.544	0.968	0.460	2.040	0.988	0.469	2.082
**Acute myocardial infarction**	0.552	0.055	5.526	0.000	0.000	-	0.833	0.180	3.866	1.393	0.292	6.646
**Heart failure**	1.436	0.116	17.801	0.000	0.000	-	2.493	0.360	17.256	0.000	0.000	-
**Anxiety-depression**	0.857	0.142	5.180	0.000	0.000	-	2.368	0.916	6.121	0.352	0.117	1.060
**ACT ≤ 19 (Not controlled)**	0.581	0.124	2.719	8.415	0.447	158.459	1.235	0.478	3.191	0.929	0.359	2.407
**Chronic OCS** use	2.609	0.257	26,49	0.951	0.038	23.832	0.704	0.158	3.140	1.162	0.300	4.504
**LAMA use**	**11.297***	2.009	63.54	0.522	0.046	5.876	1.823	0.735	4.519	**0.277****	0.105	0.731
**Nasal CS use**	**0.159****	0.048	0.52	0.924	0.155	5.507	1.453	0.740	2.854	1.497	0.771	2.906
**Antileukotriens**	3.742	0.318	43.987	0.000	0.000	-	1.597	0.592	4.310	0.668	0.251	1.778
**Asthma severity grade**												

**GINA (step 1)**	0.000	0.000	-	0.426	0.014	12.965	0.670	0.164	2.740	3.783	0.917	15.598
**GINA (step 2)**	0.695	0.106	4.579	0.094	0.002	4.296	1.000	0.294	3.401	1.350	0.391	4.663
**GINA (step 3)**	1.175	0.140	9.840	0.034	0.000	2.871	0.590	0.149	2.341	1.770	0.451	6.950
**GINA (step 4)**	**0.048***	0.004	0.637	0.087	0.002	4.728	1.522	0.375	6.169	2.235	0.544	9.188

*=p<0.05, **=p<0.01, ***=p<0.001. Bold font highlights statistically significant results. LB: lower bound, UB: upper bound; FVC: forced vital capacity; FEV_1: forced expiratory capacity in the first second; FRC: Functional residual capacity; CRSwNP: Chronic Rhinosinusitis with nasal polyps; LAMA: long-acting muscarinic antagonist; GINA: Global Initiative for Asthma; Obesity= BMI≥30.

Supplementary Table S3: Multinomial LRM of variables comparing each group of patients stratified according to serum Total IgE and T2 inflammatory phenotype

**Characteristic**	*$T2_{Low}$-IgE_{Low} vs.$T2_{High}$-IgE_{high}*			*$T2_{High}$-IgE_{High} vs. $T2_{Low}$-IgE_{Low}*			*$T2_{Low}$-IgE_{Low} vs.$T2_{low}$-IgE_{high}*			*$T2_{low}$-IgE_{high} vs. $T2_{Low}$-IgE_{Low}*			*$T2_{low}$-IgE_{low} vs. $T2_{High}$-IgE_{low}*			*$T2_{High}$-IgE_{low} vs. $T2_{low}$-IgE_{low}*		
	OR	LB	UB	OR	LB	UB	OR	LB	UB	OR	LB	UB	OR	LB	UB	OR	LB	UB
**Gender** *(male)*	0.274	0.072	1.046	3.651	0.956	13.94	0.150	0.021	1.057	6.676	0.946	47.09	0.464	0.120	1.792	2.155	0.558	8.321

**BMI**	**1.153***	1.016	1.309	0.867*	0.764	0.984	1.120	0.903	1.388	0.893	0.720	1.107	1.086	0.963	1.225	0.921	0.817	1.039
**Age at asthma onset**	**1.044****	1.015	1.074	0.958**	0.931	0.985	1.023	0.977	1.072	0.977	0.933	1.023	**1.035***	1.006	1.065	0.966*	0.939	0.994
**FE phenotype**	0.430	0.104	1.785	2.324	0.560	9.640	**0.127***	0.016	0.996	**7.866***	1.004	61.635	0.488	0.118	2.012	2.051	0.497	8.465
**Past smoker**	0.565	0.140	2.275	1.770	0.440	7.125	0.212	0.030	1.519	4.723	0.658	33.883	0.400	0.100	1.599	2.498	0.625	9.978
**Rhinitis**	**0.317***	0.106	0.948	**3.151***	1.055	9.410	**0.117***	0.017	0.821	**8.529***	1.218	59.723	0.349	0.117	1.038	2.866	0.963	8.530
**emphysema**	**3.984***	0.995	15.948	**0.251***	0.063	1.005	1.238	0.145	10.556	0.808	0.095	6.893	3.964	0.958	16.408	0.252	0.061	1.044
**OSAS**	0.601	0.020	18.309	1.665	0.055	50.768	0.131	0.003	5.796	7.634	0.173	337.810	0,501	0.022	11.548	1.996	0.087	46.019
**GERD**	0.735	0.228	2.368	1.360	0.422	4.379	0.510	0.081	3.217	1.962	0.311	12.381	0.757	0.240	2.390	1.321	0.418	4.169

**Obesity**	0.409	0.084	1.986	2.445	0.504	11.868	0.216	0.020	2.342	4.629	0.427	50.194	0.516	0.114	2.330	1.938	0.429	8.755
**Arterial hypertension**	0.940	0.314	2.816	1.063	0.355	3.185	0.931	0.149	5.807	1.074	0.172	6.694	0.841	0.279	2.534	1.189	0.395	3.585
**Heart failure**	**7.988E8***	1.062E8	6.010E9	**1.252E-9***	1.252E-9	1.252E-9	2.891E8	0.1	5.78E-8	1.273E-9	1.273E-9	1.273E-9	2.052	0.273	15.438	0.487	0.065	3.666
**Anxiety-depression**	1.869	0.391	8.939	0.535	0.112	2.560	**4.329E7***	9.050E7	2.071E8	**8.497E-9***	0.000	-	0.497	0.119	2.080	2.011	0.481	8.411
**Poor control**	**3.454***	1.162	10.268	**0.290***	0.097	0.861	0.328	0.051	2.118	3.046	0.472	19.644	0.398	0.138	1.150	2.513	0.870	7.262
**FEV$_1$ (L)**	1.567	0.588	4.176	0.638	0.239	1.701	1.696	0.322	8.928	0.590	0.112	3.105	1.401	0.524	3.747	0.714	0.267	1.909
**FRC (%pred)**	1.008	0.984	1.032	0.992	0.969	1.016	1.001	0.964	1.039	0.999	0.962	1.037	**1.028***	1.004	1.054	0.972*	0.949	0.996
**GINA**	0.605	0.357	1.023	1.654	0.977	2.799	0.762	0.325	1.787	1.313	0.560	3.078	0.623	0.368	1.054	1.606	0.949	2.716
**Chronic OCS** use	0.813	0.134	4.937	1.229	0.203	7.461	0.398	0.023	6.766	2.514	0.148	42.760	1.569	0.226	10.905	0.637	0.092	4,.428

**LAMA use**	**9.873****	2.411	40.435	**0.101****	0,025	0.415	5.710	0.604	53.949	0.175	0.019	1.654	3.358	0.885	12.741	0.298	0.078	1.130
**Nasal CS use**	**0.280***	0.101	0.777	**3.567***	1.287	9.890	0.557	0.103	3.006	1.796	0.333	9.701	**0.278***	0.100	0.770	**3.598***	1.299	9.965
**antileukotrienes**	1.853	0.328	10.457	0.540	0.096	3.045	1.786E7	0.1	3.57E-7	2.060E-8	0.000	-	1.213	0.223	6.582	0.825	0.152	4.476

**Characteristic**	*$T2_{low}$-IgE_{high} vs. $T2_{High}$-IgE_{Low}*			*$T2_{High}$-IgE_{Low} vs. $T2_{low}$-IgE_{high}*			*$T2_{low}$-IgE_{high} vs. $T2_{High}$-IgE_{High}*			*$T2_{High}$-IgE_{High} vs. $T2_{Low}$-IgE_{High}*			*$T2_{High}$-IgE_{low} vs. $T2_{High}$-IgE_{High}*			*$T2_{High}$-IgE_{High} vs. $T2_{High}$-IgE_{low}*		
	OR	LB	UB	OR	LB	UB	OR	LB	UB	OR	LB	UB	OR	LB	UB	OR	LB	UB
**Gender** *(male)*	3.098	0.568	16.898	0.323	0.059	1.761	1.829	0.350	9.541	0.547	0.105	2.853	0.590	0.265	1.314	1.694	0.761	3.773
**BMI**	0.970	0.799	1.177	1.031	0.850	1.252	1.030	0.849	1.249	0.971	0.801	1.178	1.062	0.968	1.165	0.942	0.859	1.033
**Age at asthma onset**	1.011	0.970	1.054	0.989	0.948	1.031	1.020	0.980	1.063	0.980	0.941	1.021	1.009	0.991	1.027	0.991	0.974	1.009
**FE phenotype**	3.835	0.662	22.227	0.261	0.045	1.512	3.385	0.622	18.432	0.295	0.054	1.609	0.883	0.354	2.199	1.133	0.455	2.822

**Past smoker**	1.891	0.369	9.686	0.529	0.103	2.709	2.669	0.535	13.318	0.375	0.075	1.870	1.411	0.616	3.236	0.709	0.309	1.625
**Rhinitis**	2.976	0.497	17.798	0.336	0.056	2.010	2.707	0.469	15.623	0.369	0.064	2.132	0.910	0.428	1.933	1.099	0.517	2.336
**enphisema**	3.203	0.414	24.762	0.312	0.040	2.413	3.219	0.449	23.078	0.311	0.043	2.227	1.005	0.293	3.449	0.995	0.290	3.415
**OSAS**	3.824	0.337	43.456	0.261	0.023	2.971	4.585	0.399	52.651	0,218	0,019	2,505	1,199	0.202	7.124	0.834	0.140	4.957
**GERD**	1.486	0.287	7.695	0.673	0.130	3.487	1.443	0.285	7.294	0.693	0.137	3.504	0.971	0.423	2.232	1.030	0.448	2.367
**Obesity**	2.388	0.287	19.880	0.419	0.050	3.486	1.893	0.235	15.280	0.528	0.065	4.262	0.793	0.258	2.433	1.261	0.411	3.870
**Arterial hypertension**	0.903	0.171	4.776	1.108	0.209	5.862	1.010	0.195	5.233	0.991	0.191	5.134	1.118	0.519	2.412	0.894	0.415	1.928
**Heart failure**	2.611E-9	2.611E-9	2.611E-9	1.409E8	0.000	-	1.017	1.017	1.017	0.984	0.000	-	8.93E7	3.893E8	3.893E8	2.569E-9	2.569E-9	2.569E-9
**Anxiety-depression**	4.225E-9	4.225E-09	4.225E-09	8.706E7	2.975E7	2.548E8	1.588E-8	1.588E-08	1.588E-08	2.317E7	2.317E7	2.317E7	**3.758***	1.284	10.995	**0.266***	0.091	0.779
**Poor control**	1.212	0.223	6.598	0.825	0.152	4.492	0.882	0.167	4.647	1.134	0.215	5.975	0.728	0.338	1.567	1.374	0.638	2.960

**FEV_1 (L)**	0.826	0.185	3.683	1.210	0.272	5.393	0.924	0.212	4.034	1.082	0.248	4.725	1.118	0.606	2.063	0.894	0.485	1.650
**FRC (%pred)**	1.027	0.994	1.062	0.974	0.942	1.006	1.006	0.975	1.039	0.994	0.962	1.026	**0.980***	0.965	0.995	**1.020***	1.005	1.036
**GINA**	0.818	0.379	1.764	1.223	0.567	2.640	0.794	0.371	1.697	1.260	0.589	2.694	0.971	0.689	1.368	1.030	0.731	1.452
**Chronic OCS use**	3.945	0.262	59.449	0.253	0.017	3.819	2.045	0.157	26.667	0.489	0.038	6.376	0.518	0.117	2.303	1.929	0.434	8.573
**LAMA use**	0.588	0.075	4.629	1.700	0.216	13.384	1.729	0.229	13.034	0.578	0.077	4.360	**2.940***	1.072	8.060	**0.340***	0.124	0.933
**Nasal CS use**	0.499	0.107	2.321	2.003	0.431	9.310	0.504	0.112	2.258	1.986	0.443	8.902	1.009	0.495	2.054	0.991	0.487	2.019
**antileukotrienes**	2.50E-08	2.50E-08	2.50E-08	1.473E7	0.000	-	3.82E-08	3.82E-08	3.82E-08	9.636E6	0.000	-	1.528	0.556	4.198	0.654	0.238	1.797

*=p<0.05, **=p<0.01, ***=p<0.001. Bold font highlights statistically significant results. LB: lower bound, UB: upper bound. FE: frequent exacerbator (≥2 exacerbation/year); OSAS: obstructive sleep apnoea syndrome; GERD: gastroesophageal reflux disease; FEV_1: forced expiratory capacity in the first second; FRC: Functional residual capacity; GINA: Global Initiative for Asthma; LAMA: long-acting muscarinic antagonist; Obesity= BMI≥30.
